# Characterization of *gyrA* and *parC* mutations in ciprofloxacin-resistant *Pseudomonas aeruginosa* isolates from Tehran hospitals in Iran

**Published:** 2018-08

**Authors:** Rosetta Moshirian Farahi, Ahya Abdi Ali, Sara Gharavi

**Affiliations:** 1 Department of Microbiology, Alzahra University, Tehran, Iran; 2 Department of Biotechnology, Alzahra University, Tehran, Iran

**Keywords:** *Pseudomonas aeruginosa*, Fluoroquinolones, *GyrA*, *ParC*, Ciprofloxacin resistance

## Abstract

**Background and Objectives:**

*Pseudomonas aeruginosa*, a major cause of several infectious diseases, has become a hazardous resistant pathogen. One of the factors contributing to quinolone resistance in *P. aeruginosa* is mutations occurring in *gyrA* and *parC* genes encoding the A subunits of type II and IV topoisomerases, respectively, in quinolone resistance determining regions (QRDR) of the bacterial chromosome.

**Materials and Methods:**

Thirty seven isolates from patients with burn wounds and 20 isolates from blood, urine and sputum specimen were collected. Minimum Inhibitory Concentrations (MICs) of ciprofloxacin were determined by agar diffusion assay. Subsequently, QRDRs regions of *gyrA* and *parC* were amplified from resistant isolates and were assessed for mutations involved in ciprofloxacin resistance after sequencing.

**Results:**

Nine isolates with MIC≥8 μg/ml had a mutation in *gyrA* (Thr83→Ile). Amongst these, seven isolates also had a mutation in *parC* (Ser87→ Leu or Trp) indicating that the prevalent mutation in *gyrA* is Thr83Ile and Ser87Leu/Trp in *parC*. No single *parC* mutation was observed.

**Conclusion:**

It seems that mutations in *gyrA* are concomitant with mutations in *parC* which might lead to high-level ciprofloxacin resistance in *P. aeruginosa* isolates from patients with burn wounds and urinary tract infections.

## INTRODUCTION

A growing population of multidrug resistant bacteria has emerged as the “post antibiotic era” of infectious diseases. One prominent example is *Pseudomonas aeruginosa* which has been the focus of therapeutic challenges. This ubiquitous organism exists in many diverse environments, and can be isolated from various living sources. The ability of *P. aeruginosa* to survive in harsh conditions and endure stress has allowed the organism to persist in both community and hospitals. *P. aeruginosa* is commonly responsible for nosocomial infections in ICUs, including surgical site, urinary tract, pneumonia and bloodstream, eye, ear, nose and throat infections (1–4).

Due to the low permeability of its cell wall, *P. aeruginosa* is intrinsically resistant to most antibiotics as a result of decreased intracellular drug concentration caused by decreased uptake or increased efflux pumps expression (5–6).

Principal mechanisms of bacterial resistance to quinolones are modification of the target site (DNA gyrase) and reduction of intracellular concentration of quinolones due to mutations in the regulatory genes *mexR* and *nfxB* (7). In *P. aeruginosa*, resistance to quinolones is also often mediated by mutations in regulatory genes leading to upregulation of different efflux pumps systems (8–10). Mutations in plasmid-mediated quinolone resistance genes (pmqr) in *P. aeruginosa* have also been reported (11, 12) and attribute to high levels of resistance albeit at a lower scale compared to mutations in the *qrdr* region of the bacterium. Frequently, these resistance genes are acquired from other organisms via plasmids, transposons, bacteriophages or integrons (13).

Fluoroquinolones are an important class of wide spectrum antibacterial agents; an example is ciprofloxacin which has emerged as one of the most effective antibiotics against *P. aeruginosa* (14). Fluoroquinolones target DNA gyrase (topoisomerase II) and topoisomerase IV, which are vital in replication of bacterial DNA. DNA gyrase consists of A2 and B2 subunits encoded by the *gyrA* and *gyrB* genes. Topoisomerase IV is encoded by *parC* and *parE* subunits (15–17).

Alterations in QRDR in both *gyrA* and *parC* genes are now known to play an integral role in quinolone resistance in *P. aeruginosa* (18–20).

The aim of this study was to find possible mutations in *gyrA* of DNA gyrase and *parC* of topoisomerase IV in ciprofloxacin-resistant clinical isolates of *P. aeruginosa*. The correlation between these mutations and minimal inhibitory concentrations (MICs) of ciprofloxacin-resistance was also determined.

## MATERIALS AND METHODS

### Bacterial isolates.

Thirty seven isolates were obtained from patients with burn wounds admitted to Shahid Mottahari Burn Hospital and 20 isolates collected from blood, urine and sputum specimen at Shahid Rajaei Burn Hospital (2009–2013) in Tehran, Iran. Resistance to ciprofloxacin was evaluated by the Kirby-Bauer test (21). *P. aeruginosa* ATCC 27853, *E. coli* ATCC 25922 and *S. aureus* ATCC 25932 were used as susceptibile controls and compared to CLSI references (22–24). MIC of ciprofloxacin was measured by microdilution in ciprofloxacin-resistant isolates.

### PCR amplification and DNA sequencing.

DNA was extracted from ciprofloxacin-resistant isolates by the SET buffer method (25). QRDR amplification of *gyrA* and *parC* from resistant isolates was carried out using specific primers: *gyrA*-1 (5′-GTGTGCTTTATGCCATGAG-3′) and *gyrA*-2 (5′-GGTTTCCTTTTCCAGGTC-3′) for the amplification of 287 bp of the fluoroquinolone resistance-determining region of the *gyrA* gene and *parC*-1 (5′-CATCGTCTACGCCATGAG-3′) and *parC*-2 (5′-AGCAGCACCTCGGAATAG-3′) were used to amplify 267 bp of the fluoroquinolone resistance-determining region of *parC* as previously reported (10). In the design of *parC* amplification, the annealing temperature was increased to 59°C and for some samples up to 60°C. PCR enhancer was also added to augment the efficiency of PCR (26, 27). Amplified products were then separated using 1.5% agarose gels and PCR products were sequenced (Bioron, Germany).

### Analysis of DNA sequences.

DNA sequences obtained using forward and reverse primers were processed by Bioeditor program using pairwise alignment. The sequence of each of sample was compared with *P. aeruginosa* PAO1 sequence. The sequences were multiple aligned by Clustal W2 (http://www.ebi.ac.uk/Tools/msa/clustalw2/) in order to detect mutations. Nucleotide Sequences were translated by Expasy Bioinformatics Resource Portal (http://web.expasy.org/translate/) then compared with *P. aeruginosa* PAO1 protein sequence using Clustal W2 to find changes in amino acids sequences.

## RESULTS

**Fig. 1.**
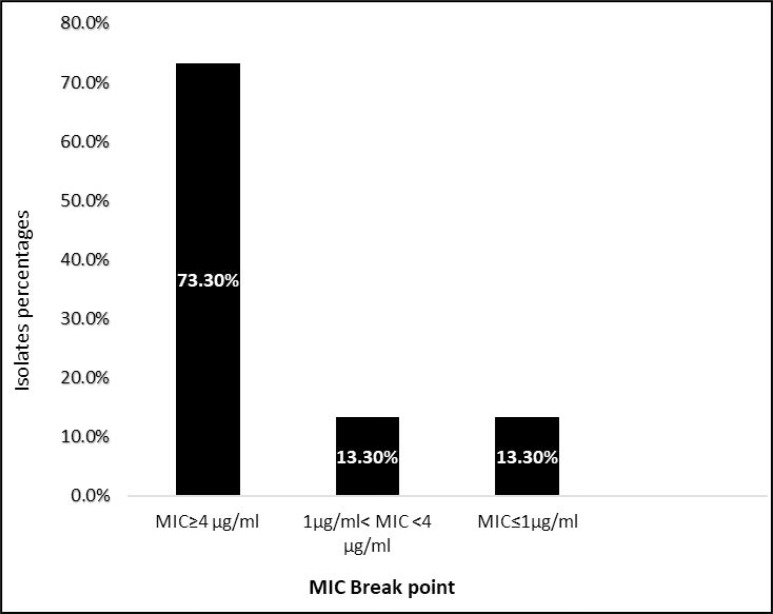
Ciprofloxacin sensitivity test using Kirby-Bauer test

Among 57 isolates, 41.37% showed resistance and 15.51% showed intermediate susceptibility to ciprofloxacin as detected by disc diffusion test (Table 1). MICs of ciprofloxacin were measured for 30 isolates (Fig. 1) of which 22 isolates were resistant (MIC >4 μg/ml).

**Table 1.** Point mutations in resistant isolates and their susceptibility to ciprofloxacin

**No.**	**Isolate name**	***gyrA* mutation**	**Silent mutation in *gyrA***	***parC* mutation**	**Silent mutation in *parC***	**MIC μg/ml**	**Diameter of inhibitory zone (mm)**	**Mutation in both genes**
1	B1	Thr ➡ Ile	_	Ser ➡ Leu	Ala ➡ Ala	32	12	Yes
		ACC83ATC		TCG87TTG	GCT115GCG			
2	B14	Thr ➡ Ile	_	Ser ➡ Leu	Ala ➡ Ala	64	13	Yes
		ACC83ATC		TCG87TTG	GCT115GCG			
3	B25	Thr ➡ Ile	_	Ser ➡ Leu	Ala ➡ Ala	64	10	Yes
		ACC83ATC		TCG87TTG	GCT115GCG			
4	B32	Thr ➡ Ile	_	Ser ➡ Leu	Ala ➡ Ala	64	13	Yes
		ACC83ATC		TCG87TTG	GCT115GCG			
5	B38	Thr ➡ Ile	_	Ser ➡ Leu	Ala ➡ Ala	32	13	Yes
		ACC83ATC		TCG87TTG	GCT115GCG			
6	B48 sensitive	_	_	_	Ala ➡ Ala	1	30	No
				Ser ➡ Leu	GCT115GCG			
7	B50	Thr ➡ Ile	_	TCG87TTG	Ala ➡ Ala	16	11	Yes
		ACC83ATC			GCT115GCG			
8	S2	Thr ➡ Ile	Val ➡ Val	_	_	32	10	_
		ACC83ATC	GTA103GTC					
			Ala ➡ Ala					
			GCA118GCG					
			Ala ➡ Ala					
			GCG136GCC					
9	S4	Thr ➡ Ile	Val ➡ Val	_	_	32	11	_
		ACC83ATC	GTA103GTC					
			Ala ➡ Ala					
			GCA118GCG					
			Ala ➡ Ala					
			GCG136GCC					
10	S14	_	His ➡ His	_	Ala ➡ Ala	8	15	No
			CAC132CAT		GCT115GCG			
11	S20	Thr ➡ Ile	_	Ser ➡ Trp	Ala ➡ Ala	64	10	Yes
		ACC83ATC		TCG87TGG	GCT115GCG			

Amongst the 57 isolates, 30 isolates were selected for the MIC test based on CLSI principles. Of the 30 clinical isolates, 22 isolates (73.3%) were resistant, 4 isolates (13.3%) showed intermediate susceptibility to CIP and 4 isolates (13.3%) were susceptible to CIP.

PCR amplification of *gyrA* and *parC* genes were carried out using DNA from 22 ciprofloxacin resistant isolates of which 10 were selected for *gyrA* sequencing and 8 for *parC* sequencing. Finally, in order to detect the correlation between resistance and *gyrA* and *parC* mutations, the results of sequencing were analyzed (Figs. 3 and 4).

**Fig. 2.**
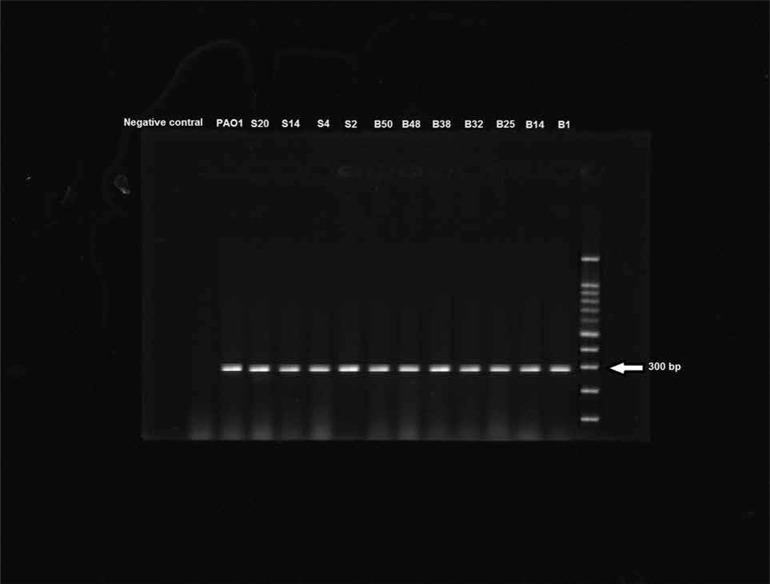
PCR products amplified with *gyrA* specific primers and electrophoresed on agarose gel

Amplification of *gyrA* resulted in specific bands of 300 bp (Fig. 2). *P. aeruginosa* strain PAO1 was used as a control for the presence of *qrdr* region, isolate B48 which was sensitive was used for comparison and a negative control was used as contamination control.

## DISCUSSION

Due to the growing number of antibiotic resistant bacteria, the significance of this resistance in MDR *P. aeruginosa* strains must be taken into greater consideration. The elucidation of the mechanisms leading to this resistance is one of the key factors involved in the treatment of hospital patients with more narrow spectrum, target specific and modified antibiotics (10).

In this study, resistance to ciprofloxacin was 41.37% which is close to the report by Tohidpour and colleagues in Tehran (35%) (28) but lower than the rates reported by Saderi and coworkers’ study in Tehran (55%) (29) and another report of Nouri and coworkers from Tabriz, Iran (30). In addition, results from the study by Lu and coworkers showed resistance of *P. aeruginosa* to ciprofloxacin in Asia-Pacific region from 2009 to 2010 was around 44.4% (31).

However, the resistance to ciprofloxacin obtained in this study was higher compared to reports from Canada (27%) (32) and also higher than published data from USA (33.1%) (33).

Statistics indicate that different resistance patterns exist among various regions. The magnitude of antibiotic use might contribute to the variety of antibiotic resistance range; therefore, elevated resistance rate in Asia might be associated with the notably increased use of antibiotics in the area (29, 31).

**Fig. 3.**
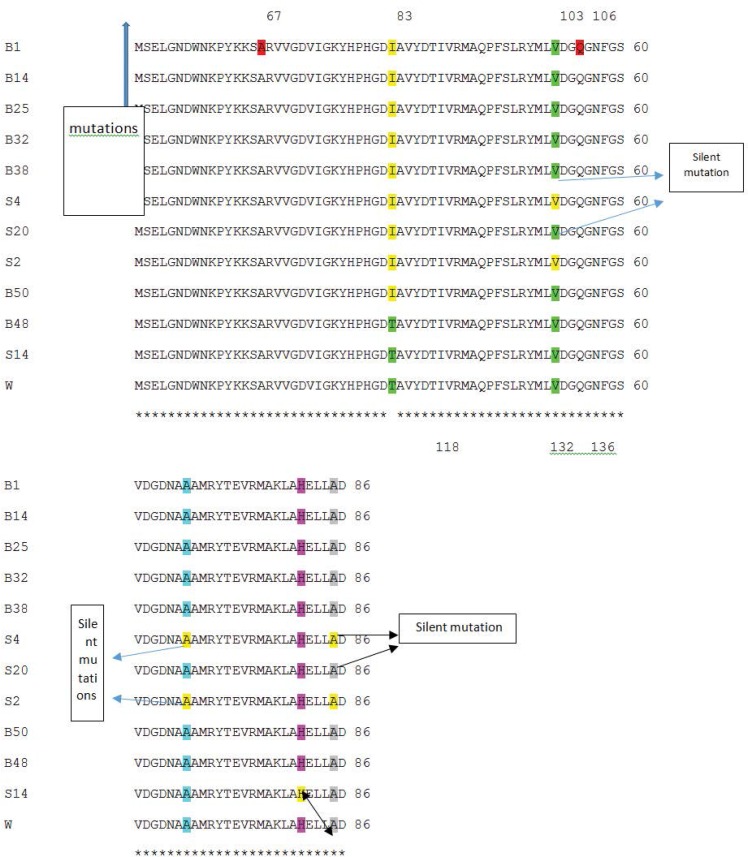
Comparison of *gyrA* PCR products sequences with *P. aeruginosa* PAO1 using CLUSTAL 2.1 multiple sequence alignment.

**Fig. 4.**
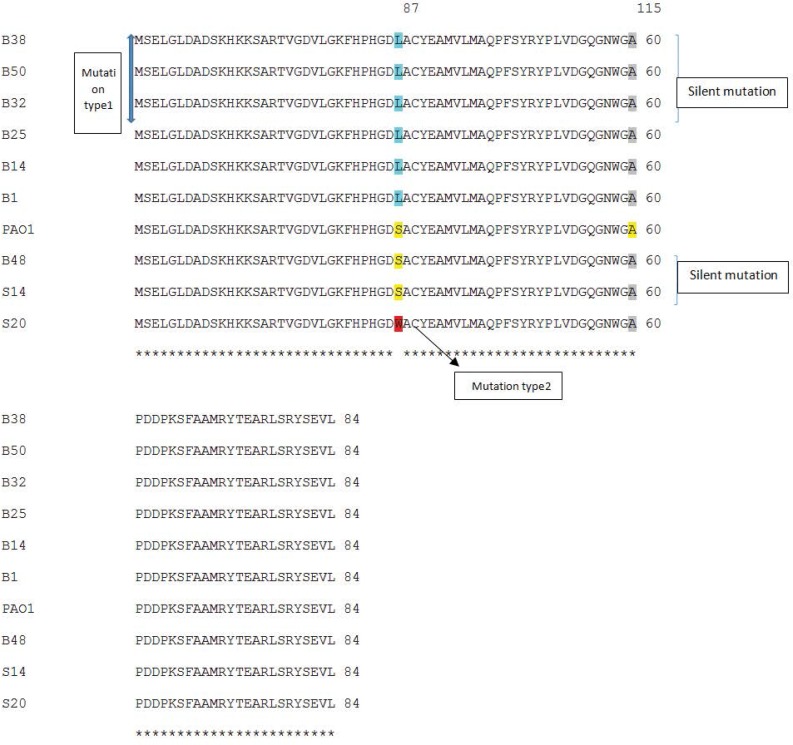
Comparison of *parC* PCR products sequences with *P. aeruginosa* PAO1 using CLUSTAL 2.1 multiple sequence alignment.

One of the major mechanisms involved in the development of quinolone resistance is the mutational alterations in DNA gyrase. In this study, almost 90% of resistant isolates had a *gyrA* mutation and the most common was the conversion of threonine which is a polar amino acid to non-polar isoleucine amino acid at codon 83. Moreover, high-level resistance is usually associated with the presence of both *gyrA* and *parC* mutations simultaneously and one of the frequent mutation in *parC* changed serine 87 to leucine and more importantly, to tryptophan (Table 1, Figs 3 and 4). Pasca and coworkers have reported an overwhelming percentage of *gyrA* and *parC* mutations as causes for fluoroquinolone resistance in *P. aeruginosa* isolates from Northern Italian hospitals (34).

Kulberge and her colleagues have shown that single mutations in *gyrA* or *gyrB* caused low-level resistance whereas high-level resistant mutants had double mutations in *gyrA* and *parC*, *parE*, *nfxB* or unknown genes (16).

An evaluation of samples collected in this report from urine, burn wounds and sputum, show high level resistance with MIC>8 μg/ml and mutations in *gyrA* and *parC* in the isolates.

In the present study, among 8 resistant isolates, 7 had both *gyrA* and *parC* mutations causing relatively high level resistance (MIC≥16 μg/ml). The resistance of 1 isolate (S14) with only silent mutations was comparatively lower than the other resistant mutants (MIC=8 μg/ml and inhibitory zone diameter of 15 mm). It can hence be concluded that other resistance mechanisms except mutations in *gyrA* and *parC* can be held responsible for this low-level resistance (Table 1).

The alteration of polar threonine (Thr) to the non-polar and highly hydrophobic isoleucine (Ile) does not occur in active site of enzyme; thus the enzyme is able to maintain its function. This mutation is likely to influence the gyrase- quinolone interaction by loss of essential enzyme- drug contacts or conformational modifications that may ultimately result in antibiotic resistance. The presence of *gyrA* mutation in all resistant isolates endorses the fact that DNA gyrase is pivotal target enzyme in ciprofloxacin resistance in *P. aeruginosa* (35–37).

This data substantiates the experiment conducted by Higgins and coworkers in 2003 indicating that the main mechanism of fluoroquinolone resistance in *P. aeruginosa* is mediated mainly through mutations in *gyrA* and mutations in *parC* genes are subsidiary (38). Reports by Salma and coworkers (39) in Lebanon also substantiate our results and other studies (27, 36) that *P. aeruginosa* resistant mutants with sole *parC* mutations have not been detected.

## CONCLUSION

Results from this study and validation from previous research postulate that *gyrA* mutations are the major mechanism of resistance to fluoroquinolone for clinical strains of *P. aeruginosa* and demonstrate that DNA gyrase encoding gene, *gyrA* is the primary target for fluoroquinolone and further mutations in *parC* could lead to a higher level of quinolone resistance. This is the first report of *parC* mutations detected in addition to *gyrA* mutations reported earlier in resistant *P. aeruginosa* isolates from Tehran, Iran.
